# Lifelong robbery victimisation and mental disorders at age 18 years: Brazilian population-based study

**DOI:** 10.1007/s00127-018-1488-z

**Published:** 2018-02-17

**Authors:** Joseph Murray, Natália Peixoto Lima, Ana Carolina Oliveira Ruivo, Andrea Ramírez Varela, Caroline Cardozo Bortolotto, Elma Izze da Silva Magalhães, Franciéle Marabotti Costa Leite, Mariana Otero Xavier, Jean-Baptiste Pingault, Seena Fazel, Gregore Iven Mielke, Luciana Anselmi, Fernando César Wehrmeister, Helen Gonçalves, Ana Maria Baptista Menezes

**Affiliations:** 1 Postgraduate Program in Epidemiology, Federal University of Pelotas, Pelotas, Rio Grande Do Sul Brazil; 2 Nursing Department, Post Graduate Program in Nursing, Federal University of Espirito Santo, Vitoria, Espírito Santo Brazil; 3 Department of Clinical, Educational and Health Psychology, University College London, London, UK; 4 Department of Psychiatry, University of Oxford, Oxford, UK

**Keywords:** Violence, Middle-income country, Mental health, Adolescence, Crime victims

## Abstract

**Purpose:**

Urban violence is a major problem in Brazil and may contribute to mental disorders among victims. The aim of this study was to assess the association between robbery victimisation and mental health disorders in late adolescence.

**Methods:**

At age 18 years, 4106 participants in the 1993 Pelotas Birth Cohort Study were assessed. A questionnaire about history of robbery victimisation was administered, the Self-Report Questionnaire was used to screen for common mental disorders, and the Mini International Neuropsychiatric Interview was used to assess major depressive disorder and generalised anxiety disorder. Cross-sectional prevalence ratios between lifetime robbery victimisation and mental disorders were estimated using Poisson regression with robust standard errors, adjusting for socioeconomic variables measured at birth and violence in the home and maltreatment measured at age 15.

**Results:**

There was a dose–response relationship between frequency of lifetime robberies and risk of mental disorders. Adolescents who had been robbed three or more times had twice the risk (PR 2.04; 95% CI 1.64–2.56) for common mental disorders, over four times the risk for depression (PR 4.59; 95% CI 2.60–8.12), and twice the risk for anxiety (PR 1.93; 95% CI 1.06–3.50), compared with non-victims, adjusting for covariates. Experiencing frequent robberies had greater impact on common mental disorders than experiencing an armed robbery. Population attributable fractions with regard to robbery were 9% for common mental disorders, 13% for depression, and 8% for anxiety.

**Conclusions:**

Robberies are associated with common mental disorders in late adolescence, independently of violence between family members. Reducing urban violence could significantly help in preventing common mental illnesses.

**Electronic supplementary material:**

The online version of this article (10.1007/s00127-018-1488-z) contains supplementary material, which is available to authorized users.

## Introduction

In 1996, the World Health Organization declared that violence was a major and growing public health problem, accounting for more than 1 million deaths worldwide per year [1]. Violence is defined as the use of physical strength, power or any threat that can result in physical and psychological harm [2]. The highest rates of serious violence, as indicated by homicide rates, are found in sub-Saharan Africa and in Latin America and the Caribbean, where interpersonal violence was the leading cause of death among 15–49 year olds in 2013 [3]. As well as physical injuries, exposure to non-lethal violence has been linked to mental health problems and poorer quality of life, including impaired personal and professional relationships [4–7].

The mental health consequences of community violence, between individuals outside of the family context [8], has been less studied than other forms of violence, such as intimate partner violence [9] and child maltreatment [10, 11]; however, strong effects of community violence on posttraumatic stress disorder and externalising behaviour have been reported [12]. Most studies of community violence that have considered other mental heath outcomes have examined internalising problems in a broad fashion [7], with mixed results [12], and few have examined its effects while also considering violence experienced in the home [13]. Although one meta-analysis on the effects of community violence on internalising problems found a weak association [12], co-occurring violence in the home was not taken into account, which might have biased the results [13].

Brazil has experienced an enormous rise in serious urban violence over the past three decades [14]. According to a national victimisation survey in 2011/2012, 4% of adults had been victims of robbery in a 12-month period, 2% had been threatened with a gun or knife, and 1% had been victim of a sexual offence [14]. Late adolescence (ages 16–24) is when individuals are at highest risk for robbery or theft in Brazil [15]. In the city of Pelotas in Southern Brazil, where the current study was conducted, one-fifth of individuals born in 1993 had been officially registered as a victim of crime by age 18 years, with physical injury and robbery/blackmail being the most common types of victimisation [16]. The aim of the the current study was to assess the association between lifetime robbery victimisation and common mental disorders, depression and anxiety, among 18-year-old adolescents in the 1993 Pelotas Birth Cohort Study, in southern Brazil. Additionally, we examine the population attributable fraction associated with victimisation to consider the potential benefits for mental health if robberies could be reduced in this context.

## Methods

We conducted cross-sectional analyses of data from the 1993 Pelotas Birth Cohort Study. Pelotas is a city in Southern Brazil with a population of 328,275 inhabitants and a Human Development Index of 0.739 [17]. In 1993, all hospital live births from the urban area of Pelotas (*N* = 5265) were eligible to participate in the birth cohort study. After 16 refusals, 5249 were included in the study. Follow-ups were conducted during childhood and adolescence. Details on the study’s methodology can be found elsewhere [18, 19].

This current study is based on data collected during the 18-year follow-up (*N* = 4106; 81.3% of the original cohort), which was carried out by a trained team of interviewers between September 2011 and April 2012. All participants in the cohort were invited to attend the clinic at the Postgraduate Program in Epidemiology, at the Federal University of Pelotas. This study was approved by the Research Ethics Committee of the Faculty of Medicine at the Federal University of Pelotas, and participants and/or parents/guardians received relevant information about the study and signed an informed consent form.

### Measures

Information about robbery victimisation was obtained from the following questions in personal interviews: “Considering your entire life, *have you ever been robbed*?” “[If yes] *How many times*?” and “In this robbery (or any of these robberies), was a weapon used?” We examined both the frequency of robbery victimisation and the severity of victimisation (indicated by weapon use) as the main exposure variables for this study. In the original interviews in Portuguese, participants were asked about incidents of “assalto” which can be translated as “robbery” [20]; however, it is also possible some participants interpreted “assalto” more widely, and might have included assaults, or perhaps some non-violent thefts. Thus, not all events were necessarily robberies, although according to follow-up questions, most involved the use of a weapon.

The Brazilian version of the Self-Report Questionnaire (SRQ-20) was used to assess common mental disorders, which is a screening tool to investigate non-psychotic symptoms in the last month, especially depression and anxiety. The WHO suggests the SRQ-20 is suitable for studies with community samples in developing countries. The instrument includes four questions about physical symptoms and 16 questions about emotional symptoms, with dichotomous (yes or no) answers. Following a validation study in Brazil, male participants were classified as positive for common mental disorders if they answered positively to six or more questions (sensitivity 89% and specificity 81%), and women were classified positive for common mental disorders if they answered positively to eight or more questions (sensitivity 86% and specificity 77%) [21].

The Mini International Neuropsychiatric Interview instrument (MINI) was used to diagnose major depressive disorder and generalised anxiety disorder according to DSM-IV criteria [22], and has been validated previously for the Brazilian population [23]. It is a short standardized interview (15–30 min) designed to be used both for outcome-tracking in clinical practice and in epidemiological studies.

A priori, we included exposure to violence and maltreatment in the home as control variables. Violence and maltreatment in the home were measured in the age 15 follow-up of the cohort, based on five questions in a confidential questionnaire that were previously found to associate with depression at age 18 [24].

In addition, the following variables measured in the perinatal assessment were considered as possible additional confounding variables for inclusion in the final model: sex, maternal age at birth (complete years categorized as 13–19, 20–29, 30–39, and 40 or more), maternal education at birth (complete years of study, categorized as 0–8 and 9 or more), family income at birth (categorized in quintiles), mother living with a partner at birth (yes or no). Maternal common mental disorder (yes or no) measured in the 11-year follow-up was also considered as a possible confounding variable, as well as the adolescent’s skin colour (self-reported at age 15 years and subsequently categorized as white/black/brown/other).

### Analyses

Statistical analyses were performed in STATA version 14.0. Bivariate analyses were used to examine the associations between sociodemographic characteristics, robbery victimisation, and mental health using the heterogeneity Chi square test and the Chi square test for linear trend in the case of ordinal variables. Possible confounding variables were identified as those associated (*p* < 0.20) with both the exposure and at least one outcome variable, and were included in the final multivariate analysis models. Poisson regression with robust standard errors was used to obtain adjusted effect estimates (including confounding factors) [25]. The *p* value for statistical significance was < 0.05 in the final model. The main analyses were focused on the number of robbery victimisations, but we also stratified additional analyses of the effects of robbery on common mental disorders according to whether or not a weapon had been used. There was no interaction between sex and robbery exposure in predicting the mental health outcomes (all *p* ≥ 0.05), which is why analyses were not stratified by sex. Population attributable fractions (PAF) were calculated using the following equation: ${\text{PAF}}=\frac{{p({\text{PR}} - 1)}}{{p({\text{PR}} - 1)+1}}$, where *p* is the proportion of the sample exposed to robbery, and PR is the fully adjusted prevalence ratio representing the increased risk of a mental health outcome caused by exposure to at least one robbery.

## Results

At the 18-year-old follow up, 4106 adolescents participated in the study, corresponding to a follow-up rate of 81.3% (including in the denominator participants at age 18 and 164 deaths that had occurred mostly in early infancy). Among the 4106 interviewed participants, 50.9% were females, and the majority (64.2%) were white. At age 18 years, 27.4% of the participants reported they had been robbed at least once in their lifetime, and 18.5% reported having been victim of at least one armed robbery. In terms of frequency, 17.8% of youth had been robbed once, 6.2% twice and 3.4% reported three or more lifetime robberies. With respect to common mental disorders, 27.3% screened positive, and 4.0 and 7.5% of the adolescents presented with major depressive disorder and generalised anxiety disorder, respectively (Table 1).

**Table 1:** Characteristics of participants assessed in the age 18 year follow-up of the 1993 Pelotas (Brazil) Birth Cohort Study

	*N*	%
**Perinatal measures**		
Sex		
Male	2015	49.1
Female	2091	50.9
Maternal age		
13–19	702	17.1
20–29	2186	53.3
30–39	1129	27.5
40 or more	88	2.1
Maternal education (study years)		
0–8	3053	74.5
9 or more	1046	25.5
Mother lives with partner		
No	488	11.9
Yes	3618	88.1
**Age 11–15 years**		
Skin colour		
White	2526	64.2
Black	568	14.5
Brown	697	17.7
Other	143	3.6
Maternal mental disorder		
No	2724	69.2
Yes	1210	30.8
**Age 18 years**		
Number of robbery victimisations		
Zero	2978	72.6
Once	732	17.8
Twice	253	6.2
Three or more	141	3.4
Armed robbery		
Never robbed	2978	72.6
Robbed without weapon	366	8.9
Robbed with weapon	760	18.5
Common mental disorder		
No	2983	72.7
Yes	1118	27.3
Major depressive disorder		
No	3890	96.0
Yes	163	4.0
Generalised anxiety disorder		
No	3747	92.5
Yes	305	7.5

The lifetime prevalence of robbery victimisation according to maternal and youth characteristics is shown in Table 2. Higher frequencies of robbery victimisation were reported by males, adolescents who self-reported white or other skin colour, participants with higher maternal education, and those in the higher quintiles of family income.

**Table 2:** Lifetime robbery victimisation up to age 18 years according to sociodemographic and family characteristics in the1993 Pelotas (Brazil) Birth Cohort Study

	Number of robbery victimisations			
	0	1	2	≥ 3
	*N* (%)	*N* (%)	*N* (%)	*N* (%)
**Perinatal measures**				
Sex	*p* < 0.001			
Male	1275 (63.3)	453 (22.5)	168 (8.3)	118 (5.9)
Female	1703 (81.4)	279 (13.4)	85 (4.1)	23 (1.1)
Maternal age	*p* = 0.666			
13–19	507 (72.2)	123 (17.5)	42 (6.0)	30 (4.3)
20–29	1572 (72.0)	401 (18.4)	137 (6.3)	75 (3.4)
30–39	827 (73.3)	195 (17.3)	72 (6.4)	34 (3.0)
40 or more	71 (80.7)	13 (14.8)	2 (2.3)	2 (2.3)
Maternal education	*p* < 0.001			
0–8 years	2255 (73.9)	540 (17.7)	163 (5.3)	94 (3.1)
9 or more	718 (68.7)	191 (18.3)	90 (8.6)	140 (3.4)
Mother lives with partner	*p* = 0.335			
No	352 (72.1)	79 (16.2)	36 (7.4)	21 (4.3)
Yes	2626 (72.6)	653 (18.1)	217 (6.0)	120 (3.3)
Family income at birth	*p* = 0.001			
1st quintile (poorest)	605 (77.6)	117 (15.0)	43 (5.5)	15 (1.9)
2nd quintile	677 (71.8)	180 (19.1)	55 (5.8)	31 (3.3)
3rd quintile	513 (73.0)	126 (18.0)	38 (5.4)	25 (3.6)
4th quintile	589 (72.6)	153 (18.9)	46 (5.7)	23 (2.8)
5th quintile (richest)	543 (68.1)	143 (17.9)	69 (8.7)	42 (5.3)
**Age 11–15 years**				
Skin colour	*p* < 0.001			
White	1780 (70.5)	466 (18.5)	175 (6.9)	104 (4.1)
Black	450 (79.3)	82 (14.4)	28 (4.9)	8 (1.4)
Brown	531 (76.2)	116 (16.6)	34 (4.9)	16 (2.3)
Other	99 (69.2)	32 (22.4)	9 (6.3)	3 (2.1)
Maternal mental disorder	*p* = 0.242			
No	1958 (71.9)	497 (18.3)	164 (6.0)	103 (3.8)
Yes	885 (73.1)	207 (17.1)	84 (6.4)	34 (2.8)

Row percentages. Chi square heterogeneity tests

Examining mental health outcomes according to socioeconomic and demographic characteristics (Table 3), a higher prevalence of common mental disorders, depression and anxiety was found among female adolescents, those in poorer families, and among participants whose mothers had common mental disorders. Common mental disorders were also more common among adolescents with self-reported black/brown skin colour, and those whose mothers were younger, had lower education, and were not living with a partner. Adolescents who experienced more frequent robberies had particularly increased rates of common mental disorders, depression, and anxiety. Experiencing an armed robbery was associated with higher risk for common mental disorders than non-armed robbery.

**Table 3:** Mental health problems at age 18 years according to sociodemographic and family characteristics and lifetime robbery victimisation in the 1993 Pelotas (Brazil) Birth Cohort Study

	Common mental disorder		Major depressive disorder		Generalised anxiety disorder	
	*N* (%)	*p*	*N* (%)	*p*	*N* (%)	*p*
**Perinatal measures**						
Sex		< 0.001		< 0.001		< 0.001
Male	467 (23.2)		44 (2.2)		80 (4.0)	
Female	651 (31.2)		119 (5.7)		225 (10.9)	
Maternal age		0.001*		0.096*		0.190*
13–19	233 (33.3)		30 (5.6)		58 (8.4)	
20–29	577 (26.4)		79 (3.7)		165 (7.7)	
30–39	283 (25.1)		42 (3.8)		77 (6.9)	
40 or more	24 (27.3)		3 (3.4)		5 (5.7)	
Maternal education		< 0.001		< 0.001		0.093
0–8 years	889 (29.2)		139 (4.6)		239 (7.9)	
9 or more	225 (21.5)		24 (2.3)		65 (6.3)	
Mother lives with partner		0.023		0.032		0.607
No	154 (31.6)		28 (5.8)		266 (7.5)	
Yes	964 (26.7)		135 (3.8)		39 (8.1)	
Family income at birth		< 0.001*		< 0.001*		0.004*
1st quintile (poorest)	252 (32.3)		37 (4.8)		66 (8.5)	
2nd quintile	270 (28.7)		49 (5.3)		81 (8.7)	
3rd quintile	205 (29.2)		36 (5.2)		57 (8.2)	
4th quintile	208 (25.7)		22 (2.8)		52 (6.5)	
5th quintile (richest)	165 (20.7)		17 (2.2)		42 (5.4)	
**Age 11–15 years**						
Skin colour		< 0.001		0.143		0.087
White	621 (24.6)		100 (4.0)		174 (7.0)	
Black	185 (32.6)		18 (3.2)		45 (8.0)	
Brown	227 (32.6)		37 (5.3)		67 (9.8)	
Other	41 (28.7)		3 (2.1)		8 (5.8)	
Maternal mental disorder		< 0.001		0.042		0.045
No	660 (24.3)		97 (3.6)		186 (6.9)	
Yes	408 (33.8)		60 (5.0)		105 (8.7)	
**Age 18 years**						
Number of robbery victimisations		< 0.001*		< 0.001*		0.005
Zero	759 (25.5)		109 (3.7)		215 (7.3)	
Once	203 (27.7)		25 (3.5)		44 (6.1)	
Twice	94 (37.2)		15 (6.0)		31 (12.4)	
Three or more	62 (44.0)		14 (10.0)		15 (10.7)	
Armed robbery		< 0.001*		0.051*		0.159
Never robbed	759 (25.5)		109 (3.7)		215 (7.3)	
Robbed without weapon	100 (27.3)		14 (3.9)		22 (6.2)	
Robbed with weapon	259 (34.1)		40 (5.3)		68 (9.1)	

Chi square heterogeneity tests

* Linear trend test

The associations between robbery victimisation and mental health outcomes are shown in Table 4, both before and after adjusting for socioeconomic and demographic variables (sex, skin colour, maternal education, and family income at birth), violence in the home, and maltreatment (see the Methods section for how were selected). Even adjusting for confounders, a dose–response effect was found between the number of times participants were victims of robbery and their risk for mental health problems. For example, in adjusted analyses, the risk for common mental disorders was 19% higher among individuals who had been robbed once in their lifetime (compared to those who were never robbed), but this increased to 63% and 104% higher risk among those who had suffered two, and three or more robberies, respectively. Comparing participants who experienced three or more robberies to the no robbery group, diagnoses of depression and anxiety were 359% and 93% more likely, respectively. Comparing youth who had suffered armed robbery to the no robbery group, risk for common mental disorder, depressive disorder, and generalized anxiety disorder was 49% higher, 78% higher, and 48% higher, respectively.

**Table 4:** Prevalence ratios for mental health problems at age 18 years according to lifetime robbery victimisation in the 1993 Pelotas (Brazil) Birth Cohort Study

	*N* (%)	Common mental disorder		Major depressive disorder		Generalised anxiety disorder	
		Unadjusted	Adjusted^a^	Unadjusted	Adjusted	Unadjusted	Adjusted
		PR (95% CI)	PR (95% CI)	PR (95% CI)	PR (95% CI)	PR (95% CI)	PR (95% CI)
**Number of robbery victimisations**		*p* < 0.001	*p* < 0.001	*p* = 0.006	*p* < 0.001	*p* = 0.005	*p* < 0.001
Zero	2978 (72.6)	1.00	1.00	1.00	1.00	1.00	1.00
Once	732 (17.8)	1.09 (0.95–1.24)	1.19 (1.04–1.36)	0.94 (0.61–1.44)	0.99 (0.63–1.55)	0.84 (0.61–1.15)	1.01 (0.73–1.39)
Twice	253 (6.2)	1.46 (1.23–1.73)	1.63 (1.37–1.95)	1.62 (0.95–2.74)	2.30 (1.36–3.88)	1.70 (1.12–2.42)	2.18 (1.52–3.12)
Three or more	141 (3.4)	1.72 (1.42–2.10)	2.04 (1.64–2.56)	2.70 (1.59–4.59)	4.59 (2.60–8.12)	1.47 (0.89–2.41)	1.93 (1.06–3.50)
**Armed robbery**		*p* < 0.001	*p* < 0.001	*p* = 0.129	*p* = 0.011	*p* = 0.159	*p* = 0.024
Never robbed	2978 (72.6)	1.00	1.00	1.00	1.00	1.00	1.00
Robbed without weapon	366 (8.9)	1.07 (0.90–1.28)	1.14 (0.94–1.37)	1.06 (0.61–1.83)	1.09 (0.62–1.93)	0.84 (0.55–1.29)	1.07 (0.71–1.62)
Robbed with weapon	760 (18.5)	1.34 (1.19–1.50)	1.49 (1.31–1.68)	1.44 (1.01–2.05)	1.78 (1.22–2.60)	1.24 (0.96–1.61)	1.48 (1.12–1.97)

Poisson regression with robust variance. Adjusted for sex, skin colour, maternal education and family income at birth, and domestic violence/maltreatment up to age 15

*PR *prevalence ratio,* CI* confidence interval

**Fig. 1:**
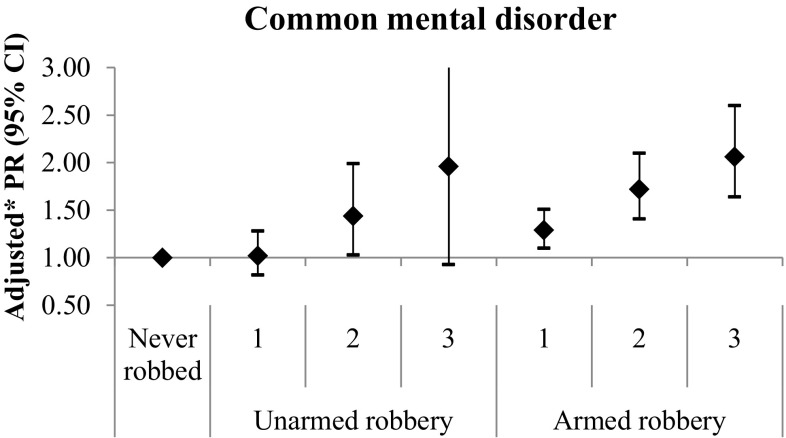
Prevalence ratio (PR) for common mental disorder at 18 years according to number of robbery victimisations, stratified according to whether or not the person had ever experienced an armed robbery. * Adjusted for sex, skin colour, maternal education and family income at birth, and domestic violence/maltreatment up to age 15. *CI* confidence interval

As one would expect, adolescents who had experienced more frequent robberies were also more likely to have experienced an armed robbery. Among youth who had experienced three or more robberies, 88% had experienced an armed robbery, compared to 74% among youth who had experienced two robberies and 61% among victims of a single robbery (*χ*^2^ = 43.15 (2); *p* < 0.001). To disentangle the effects on mental health of robbery frequency and robbery severity (weapon use), we examined the risk for common mental disorders associated with robbery frequency, stratifying by whether or not the youth had ever experienced armed robbery (in these stratified analyses with smaller numbers, only common mental disorders were analysed). Figure 1 shows adjusted prevalence ratios from these stratified analyses. The prevalence of common mental disorders increased with more frequent victimisations, even among youth who had never experienced an armed robbery; moreover, there was no evidence that this dose–response pattern was modified by the experience of an armed robbery. Hence, it appears that the frequency of robbery victimisation was more important in determining common mental disorders than whether or not adolescents had ever experienced an armed robbery, although confidence intervals are quite wide for these results.

Finally, we calculated the population attributable fraction of mental health outcomes associated with any lifetime robbery victimisation, using adjusted prevalence ratios. According to these calculations, if the adjusted prevalence ratios represent causal effects, reducing robberies in this population could, theoretically, prevent up to 9% of common mental disorders, 13% of major depressive disorders and 8% of generalized anxiety disorders.

## Discussion

The key finding in this Brazilian population-based study was that multiple robbery victimisation was associated with increased risk for common mental health disorders, anxiety and depression, even adjusting for several possible confounding variables. We discuss findings about the sociodemographic characteristics of robbery victims, possible mechanisms accounting for the effects of robbery on mental disorder, and implications for policy and practice.

Three out of ten adolescents in the current study experienced robbery by age 18 years, reflecting the high prevalence of urban violence in Brazil [14, 26, 27]. Male, white, and richer adolescents had elevated rates of robbery victimisation, as in other Brazilian studies [15, 28, 29]. The fact that males were at greater risk of robbery than females is consistent with international studies showing that, although females suffer higher rates of domestic violence and sexual crimes, males are more involved in urban violence including robbery, assault, and homicide [7, 30]. Considering that white and richer adolescents were at elevated risk of robbery, racial and socioeconomic victimisation patterns clearly depend on the type of violence being examined, given that black people and people in more disadvantaged socioeconomic groups are at much higher risk of homicide in Brazil [31, 32]. In the United States, black people are also among the main victims of homicide, accounting for more than half of all cases [33].

In the current study, robbery victimization was associated with higher rates of common mental disorders, major depressive disorder, and generalized anxiety disorder, especially when adolescents had suffered multiple events of robbery victimisation. Recent robbery, assault or other physical aggression was also associated with common mental disorders among university employees in a study in Rio de Janeiro [34], and strong associations between assaultive violence and generalised anxiety disorder and major depression were found in another study in both Rio de Janeiro and São Paulo [35]. In São Paulo, a particularly large effect of exposure to multiple types of crimes was reported in a third survey [36]. Hence, the findings of the current study, specific to robbery, are consistent with other Brazilian studies that have examined exposure to additional forms of urban violence.

Armed robbery is likely to be particularly traumatic for victims. The presence of physical violence, and experiences of terror or hopelessness during a robbery, have been associated with post-traumatic stress disorder [37]. In the current study, armed robbery also carried greater risk for common mental disorders compared to non-armed robbery. However, considering all events to age 18, it appeared that frequency of robbery was even more important in determining common mental disorders than whether or not armed robbery had ever occurred, corresponding to a cumulative risk model of trauma and psychological distress [38–40].

It was notable that the effects of robbery on mental disorders in the current study persisted even after adjusting for indicators of child maltreatment and domestic violence, as well as sociodemographic variables measured early in life. In fact, adjusted associations were actually stronger than in unadjusted analyses—a form of negative confounding [41]. Negative confounding arose because sociodemographic factors, such as family income, were positively associated with robbery, but were inversely associated with mental health disorders. Few prior studies have examined the effects of community violence on mental health while accounting for the influence of violence in the home. In one British study, Cecil et al. [13] found that exposure to community violence did not predict internalising problems after adjusting for maltreatment. The difference in findings between this and our Brazilian study might reflect lower rates of community violence in Britain, given that only multiple events of robbery victimisation were associated with mental disorders in the current study, or different measures of mental health used between the studies; also, in Britain, community violence was measured as a composite variable referring to witnessing and hearing about violence, in addition to victimisation, which was the sole focus of our Brazilian study.

Theories about the effects of community violence on mental health have largely been based on the more consolidated body of research on the effects of maltreatment on children [7, 10]. Tolan [7] discusses three main theories linking community violence and mental disorders. Trauma theories suggest that the frightening nature of violent victimisation may give rise to overactive fear conditioning and stress on the hypothalamic–pituitary–adrenal system, leading to hypervigilance and related anxiety and depression; see also Moffitt [42] on the stress-biology of these effects. By contrast, attachment theories emphasise the disruption to social relationships that can be caused by violent victimisation, rather than effects on neurobiological systems. According to an attachment perspective, victimisation in the community may highlight the inability of carers to guarantee safety and a sense of belonging in the outside world, “which acts as a trauma to undercut relationship engagement, trust, and communication” [7]. In disadvantaged neighbourhoods, violence in the form of assault and homicide exposure might also cause psychopathology as part of “an overall ecology of exceptional stress and unpredictability” [7]; however, this seems less likely with respect to robbery victimisation in the current study, which was more common among socially advantaged groups. In contrast with these theories about victimisation causing mental disorder, reverse causality is also possible. For example, mental disorders could cause victimisation if mental illness leads to increased involvement in high-risk environments, or if it causes behaviour that elicits grievances in others [43–45]. Although eliciting grievances could occur in ongoing, interpersonal relationships, or in assaults between known parties, this seems less plausible with respect to robbery, normally a quick incident conducted by strangers in an urban environment.

By calculating population attributable fractions, we estimated that up to 9% of common mental disorders, 13% of major depressive disorders, and 8% of generalized anxiety disorders among adolescents could potentially be avoided by reducing robberies. This assumes that the observed associations are causal, i.e. that reverse causality did not occur and that there is no residual confounding. Although residual confounding would reduce these estimates, even small effects on depression and anxiety may have important practical implications, given the significance of these disorders for public health. Worldwide, mental and substance use disorders are the leading cause of years lived with disability [46], and depression and anxiety are the two most important contributors. Prevention of urban violence in Brazil is an enormous challenge for justice, health, and educational systems, and may not be achieved substantially for many years. The effects of robberies and other forms of violence on mental health need addressing in this context. Police who respond to violent victimisation in the community should be aware of possible effects on mental disorders, especially for people who have experienced multiple victimisations; police training should highlight the need to relay high-risk cases for evaluation by mental health professionals. In mental health treatment settings, clinicians should consider community violence victimisation as a possible source of common mental disorders such as depression and anxiety, and not only as a source of post-traumatic stress disorder, with which urban violence has been primarily associated. Hence, screening for a history of urban violence should be routine practice in high-violence social contexts such as Brazil.

### Strengths and limitations

Our findings should be interpreted in view of the following limitations. We lacked more detailed information about the context, type and timing of the robberies reported on in this study. It would have been desirable to test whether robbery victimisation at different ages had different effects on mental disorders, but we were limited to data on lifetime robbery occurrence. For some participants, it is possible that victimisation referred to other types of crime, such as theft; however, among participants who answered positively about victimisation, two-thirds reported that a weapon was used, implying that a violent crime had definitely taken place. The self-reported lifetime robbery questions were also retrospective in nature, and although one would expect this type of traumatic event to be well remembered, prospective measures would have been preferable. Finally, we did not assess other forms of violent victimisation in the community, such as assault, or sexual offences, which might have confounded the association between robbery and the study outcomes, although the sociodemographic factors associated with robbery (male sex, higher socioeconomic position) are not likely to be positively associated with all these other types of urban violence.

Strengths of this study include the large sample and a low percentage of attrition and refusals by age 18 years. Also, there were only very small differences between individuals who had complete mental health and robbery data at age 18 compared to individuals without complete data (Online Supplementary Table). In addition, mental health outcomes were assessed with both screening and diagnostic instruments. Finally, given that this study was nested in a birth cohort, we were able to use prospectively measured variables to adjust for confounding factors from early in life.

## Conclusions

The findings of the present study suggest that robbery victimisation contributes to common mental disorders, depression and anxiety in late adolescence, and more frequent robberies carry particular risk. Greater investment in preventive interventions that decrease urban violence may help reduce mental health problems among adolescents, which represent a major public health burden. Given the elevated prevalence of violent victimisation in low- and middle-income countries such as Brazil, practitioners working with victims and clinicians in mental health services need to be attentive to the consequences of urban violence for common mental disorders such as depression and generalised anxiety, in addition to post-traumatic stress disorder, which is often associated with such events.

## Electronic supplementary material

Below is the link to the electronic supplementary material.

Supplementary material 1 (DOCX 18 KB)
