# Supplementary material for: Lifelong robbery victimisation and mental disorders at age 18 years: Brazilian population-based study
Source: Soc Psychiatry Psychiatr Epidemiol. 2018 Feb 17;53(5):487–96. doi: 10.1007/s00127-018-1488-z (PMC5908817; doi:10.1007/s00127-018-1488-z)
Supplement: Supplementary file 1 — Supplementary material 1 (DOCX 18 KB) [file 127_2018_1488_MOESM1_ESM.docx]

| **Supplementary Table.** Participants with complete data on robbery and mental health at age 18 years (N = 4047) compared to those with missing data (N = 1202), according to sociodemographic factors measured at birth in the 1993 Pelotas (Brazil) Birth Cohort Study | | | |
| --- | --- | --- | --- |
|  | **Participants with complete data  at 18 years** | **Participants with missing data  at 18 years** |  |
|  | **N (%)** | **N (%)** | ***p*** |
| **Perinatal measures** |  |  |  |
| *Sex* |  |  | *0.063* |
| Male | 1,979 (48.9) | 624 (52.0) |  |
| Female | 2068 (51.1) | 577 (48.0) |  |
| *Maternal age* |  |  | *0.412* |
| 13 - 19 | 692 (17.1) | 223 (18.6) |  |
| 20 - 29 | 2155 (53.3) | 645 (53.7) |  |
| 30 - 39 | 1111 (27.5) | 305 (25.4) |  |
| 40 or more | 88 (2.1) | 29 (2.5) |  |
| *Maternal education (study years)* |  |  | *0.278* |
| 0–8 | 3014 (74.6) | 878 (73.0) |  |
| 9 or more | 1026 (25.4) | 324 (27.0) |  |
| *Mother lives with partner* |  |  | *0.053* |
| No | 3566 (88.1) | 1034 (86.0) |  |
| Yes | 481 (11.9) | 168 (14.0) |  |
| Family income |  |  | *0.086* |
| 1st quintile (poorest) | 772 (19.4) | 259 (22.4) |  |
| 2nd quintile | 932 (23.4) | 263 (22.7) |  |
| 3rd quintile | 697 (17.5) | 192 (16.6) |  |
| 4th quintile | 798 (20.1) | 203 (17.5) |  |
| 5th quintile (richest) | 780 (19.6) | 241 (20.8) |  |
